# Mouse Organ-Specific Proteins and Functions

**DOI:** 10.3390/cells10123449

**Published:** 2021-12-08

**Authors:** Bingyun Sun, Cynthia Lorang, Shizhen Qin, Yijuan Zhang, Ken Liu, Gray Li, Zhi Sun, Ashley Francke, Angelita G. Utleg, Zhiyuan Hu, Kai Wang, Robert L. Moritz, Leroy Hood

**Affiliations:** 1 Departments of Chemistry, Simon Fraser University, Burnaby, BC V5A1S6, Canada; yijuan.bio@gmail.com (Y.Z.); kjliu@sfu.ca (K.L.); 2 Departments of Molecular Biology and Biochemistry, Simon Fraser University, Burnaby, BC V5A1S6, Canada; 3 Institute for Systems Biology, Seattle, WA 98109, USA; cynthesis3@hotmail.com (C.L.); sqin@systemsbiology.org (S.Q.); Li.Gray@systemsbiology.org (G.L.); Zhi.Sun@systemsbiology.org (Z.S.); angie.utleg@gmail.com (A.G.U.); huzy@nanoctr.cn (Z.H.); Kai.Wang@systemsbiology.org (K.W.); Robert.Moritz@systemsbiology.org (R.L.M.); 4 Departments of Computing Science, Simon Fraser University, Burnaby, BC V5A1S6, Canada; ashley.francke@gmail.com

**Keywords:** mouse proteome, organ proteome, proteome dynamics, proteome diversity, organ-specific proteins, biomarkers, brain proteome, liver proteome, kidney proteome, pancreas proteome, spleen proteome, heart proteome, skeletal muscle proteome, intestine proteome, bone proteome, eye proteome, testis proteome, lung proteome, fat proteome

## Abstract

Organ-specific proteins (OSPs) possess great medical potential both in clinics and in biomedical research. Applications of them—such as alanine transaminase, aspartate transaminase, and troponins—in clinics have raised certain concerns of their organ specificity. The dynamics and diversity of protein expression in heterogeneous human populations are well known, yet their effects on OSPs are less addressed. Here, we used mice as a model and implemented a breadth study to examine the panorgan proteome for potential variations in organ specificity in different genetic backgrounds. Using reasonable resources, we generated panorgan proteomes of four in-bred mouse strains. The results revealed a large diversity that was more profound among OSPs than among proteomes overall. We defined a robustness score to quantify such variation and derived three sets of OSPs with different stringencies. In the meantime, we found that the enriched biological functions of OSPs are also organ-specific and are sensitive and useful to assess the quality of OSPs. We hope our breadth study can open doors to explore the molecular diversity and dynamics of organ specificity at the protein level.

## 1. Introduction

Multiorgan mammals evolve in such a way that each organ has unique morphology and functions. Their molecular underpinnings have been the foundation of the diagnosis and treatment of various diseases as well as the understanding of disease etiology. Based on organ specificity, many molecular assays have been developed for monitoring organ pathological states and disease progression, such as the cardiac trophonin (cTn) test for the heart [1], alanine transaminase (ALT) and aspartate transaminase (AST) tests for the liver [2], and prostate-specific membrane antigen (PSMA) for prostate cancer [3]. However, these molecular markers are only available for a limited number of organs and diseases, and often their organ specificity is in question [4].

Several reasons hinder the research and broad applications of organ-specific proteins (OSPs). The first is their definition. Organ specificity is in fact challenging to define, particularly when we do not have a good consensus on the exact number and types of organs/tissues in humans. The narrow definition of organ specificity requires presence in one specific organ but absence in other organs. If we do not know exactly what the remaining organs are, the specificity is relative but not absolute. Second, we still cannot detect all proteins in humans. The current mapping of the human proteome is approximately 90% [5,6]. Due to their detectability, the absolute specificity of OSPs is elusive. Third, and perhaps the most pressing challenge, is the dynamics and diversity of protein expression, which compounds the first two challenges. Dynamics is the change of protein expression across time of the same individual, whereas diversity is the change across different genetic backgrounds in a population. A snapshot of a proteome cannot represent the whole. For example, many large-scale and deep proteome characterizations in humans [7,8] as well as in animal models such as mice [9,10] reported a single ensemble proteome. When studying organ specificity using such an ensemble approach, the dynamics and diversity of the proteome are averaged. However, pathological conditions, for example, are accompanied by changes in protein expression that can interfere with protein organ/tissue specificity and their use as disease diagnoses. In The Cancer Genome Atlas project (TCGA) [11], numerous novel genes were detected in ectopic organs, such as cardiac troponin I in lung cancer tissues [12].

To address the first two challenges, more tissue and organ types were examined, and more sensitive methods were employed for higher coverage and deeper penetration of the proteome. However, the mounting cost, time, and effort prevented such in-depth studies from including broad conditions and genetic backgrounds. On the other hand, genetic diversity has been recently explored on a large scale through association studies in both transcriptomes and proteomes [13,14,15], i.e., eQTLs and pQTLs. Although these studies have tended to focus on a single tissue type or on simple organisms, a large variation has been reported. Community efforts have been developed to address diversity at multiple tissue levels, such as the GTEx [16], ENCODE [17,18], TCGA [11], the Human Proteome Project (HUPO) [6] and the Human Proteome Atlas [8]. Due to the immense quantity of data, analyses often require highly experienced bioinformaticians with sufficient details on both the study and the data for meaningful results. At the proteome level, the diversity and dynamics of organ proteins are well known but are less systematically evaluated than those of transcriptomes.

As a result, none of the clinically used organ proteins such as ALT and AST are absolutely specific to their target organs [4]. Both ALT and AST have leakage expression in a number of other organs, such as the kidney. However, clinicians can use complementary assays to provide accurate diagnosis [19] once the differential expression of ALT and AST between hepatic and extrahepatic organs is known. Therefore, even though ALT and AST are not bona fide liver-specific proteins, knowledge of their blood concentrations in normal and pathological conditions still allows them to be used as popular clinical tests for liver functions. In the early pandemic of COVID-19, a lack of understanding of the precise expression of ACE2, the virus main target receptor, challenged health professionals to comprehend the mosaic pathological phenotypes observed in patients. By pooling known information together, it was shown that ACE2 is ubiquitously expressed in all tissues by transcriptomics but localized only to a number of specific cell types and body parts by direct protein measurements [20]. How diseases, such as pre-existing conditions, shift such expression patterns remains poorly addressed. Therefore, understanding and treating a novel disease will take us a long time if unprepared. Furthermore, we are probably far less equipped to diagnose and treat other diseases that are less studied than COVID.

We are urged to ask how diverse protein expression can be and to what extent information on protein localization or tissue specificity can be clinically useful tools for the diagnosis and treatment of diseases. A quantitative evaluation will be more informative than a qualitative understanding, especially in a clinical setting when both clinicians and patients demand to know the effectiveness of a particular diagnosis or treatment. To our knowledge, no quantitative evaluation has been made on the multiorgan diversity of protein expression across different genetic backgrounds, even though, for particular protein biomarkers, the sensitivity and specificity are frequently evaluated in large patient cohorts. To fill this gap, we used mice as a model and studied the proteome of 13 major organs from the commonly used BL6 strain and verified the selected organ proteomes in three other in-bred mouse strains, i.e., A/J, SJL, and NOD. We named our study a “breadth study” to differ from existing “depth studies”. Our results suggested that some but not all OSPs can vary substantially in different genetic backgrounds. We quantified such changes by robustness scores and diversity scores. Our framework can be easily scaled up to include more conditions, and the analyses can remain simple and straightforward.

## 2. Materials and Methods

Mouse strains of C57BL/6, A/J, SJL, and NOD were purchased from The Jackson Laboratory (Bar Harbor, ME, USA). Protease Inhibitor Cocktail was purchased from Sigma (St. Louis, Missouri, USA). Tris(2-carboxyethyl)phosphine (TCEP) and BCA protein assay kit were purchased from Pierce (Waltham, Massachusetts, USA), and sequence-grade trypsin was purchased from Promega (Madison, Wisconsin, USA). Rapigest and Sep-Pak C18 columns were from Waters (Milford, Massachusetts, USA), and C18 Zip tips were from Millipore (Burlington, Massachusetts, USA). All other chemicals were purchased from Fisher Scientific (Hampton, New Hampshire, USA).

### 2.1. Harvest of Animal Organs

Animal studies were approved by the Institutional Animal Care and Use Committee (IACUC) (10-00 series) of the Institute for Systems Biology (Seattle, WA, USA) with assurance from the Office of Laboratory Animal Welfare (OLAW Assurance no. A4355-01) and were accredited by the Association for Assessment and Accreditation of Laboratory Animal Care (AAALAC Accreditation no. 001363). All methods were carried out in accordance with relevant guidelines and regulations. Eight- to nine-week-old mice were fasted for 24 h prior to organ collection. Animals were euthanized and dissected immediately for targeted organs and tissues, including the liver, lung, kidney, heart, brain, small intestine, eye, pancreas, spleen, bone, skeletal muscle, fat, and testis. Sections of the excised organs were stored in 10% formalin for H&E staining in the Department of Pathology, University of Washington. Details are in the Supplementary Materials. The remaining organs were snap-frozen in liquid nitrogen and stored at −80 °C for proteomic analysis. The study was in compliance with the ARRIVE guidelines.

### 2.2. Proteomic Sample Processing

Frozen organs were pulverized in a ceramic crucible chilled with liquid nitrogen, and proteins were extracted with 2–5% SDS in PBS with 1:100 diluted Protease Inhibitor Cocktail by Precellys24 (Bertin, France) at 4 °C. The extracted protein solutions were digested by trypsin based on the filter-aided sample preparation (FASP) method [21]. Digested samples were desalted on a Sep-Pak C18 column and dried in a SpeedVac^®^ (Thermo Savant, Holbrook, NY, USA) concentrator. The Supplementary Materials describes the details of these steps.

### 2.3. Analysis of Peptides by Mass Spectrometry

The cleaned and dried peptides were reconstituted with MS loading buffer (0.1% formic acid and 1% acetonitrile) and quantified by nanodrop for peptide concentration. Two micrograms of samples at a concentration of 1 µg/µL was loaded onto nanoLC for ESI-MS/MS analysis based on published procedures [22,23]. Details are in the Supplementary Materials.

### 2.4. MS/MS Database Search, Inference, and Quantitation of Sample Proteins

MS native data files in different proprietary formats were converted to mzXML or mzML using ProteoWizard msconvert [24,25] and the spectra having fewer than six ions with intensity less than 100 were discarded [26]. MS/MS spectra were searched with Sequest [27] against a UniProt complete proteome database comprising 51,551 mouse proteins, common contaminants listed in the common repository of adventitious proteins (cRAP), and a sequence-shuffled decoy counterpart. Detailed search parameters can be found in the Supplementary Materials. The search results were processed with the Trans-Proteomic Pipeline (TPP, version 4.7) [28], including PeptideProphet [29], iProphet [30], and ProteinProphet [31], in which two–three technical and biological replicates of the same tissue were combined. A false discovery rate of less than 2.5% was used to filter the results at the protein level. We then estimated protein expression by spectral counting [32]. Proteins were normalized within each tissue by the maximum spectra count [33].

### 2.5. Analysis of Organ-Specific Proteins

We used detection breadth (DB) to describe the distribution of each protein in the sampled organs. Based on DB, we defined our organ-specific proteins (OSPs) as DB = 1, i.e., proteins detected only in one organ without specific descriptions. For the remaining proteins, we defined common (DB = all) and shared (all > DB > 2) proteins. When verifying how each protein behaved across different mouse stains, we defined the robustness score (the R score), for which detection in a distinct genetic background was scored 1. Therefore, the maximum R score in our study was 4. Because we frequently compared proteins across multiple strains or conditions, we also defined the diversity score (*D* score) that was normalized between 0 and 1 for the degree of difference between the compared individual and the whole. The *D* score is computed by

$$D=\frac{N_{unique}-N_{min}}{N_{max}-N_{min}}$$

where *N_unique_* is the number of unique entries compared across datasets, *N_min_* is the minimum possible number of unique entries, and *N_max_* is the total number of combined entries before removing the redundancy. The maximum value of the *D* score is “1”, which means that all entries are unique to each other and no overlap exists. A value less than 1 suggests that certain entries are shared by two or more datasets, i.e., decreased diversity. A value of “0” means that identical entries are among all datasets, i.e., no diversity.

### 2.6. Functional Enrichment Analysis

We used DAVID Bioinformatics Resources [34] to analyze the enriched biological functions in our datasets based on Gene Ontology Biological Processes (GO_BP_Direct/GO_BP_FAT). Using the default EASE score (a modified Fisher exact p-value that is more conservative) higher than 0.05, we obtained the enriched GO_BP terms against the total mouse genome background. Organ-specific functions were defined by the presence of only one type of organ/tissue.

## 3. Results and Discussion

### 3.1. Overall Results

**Figure 1:**
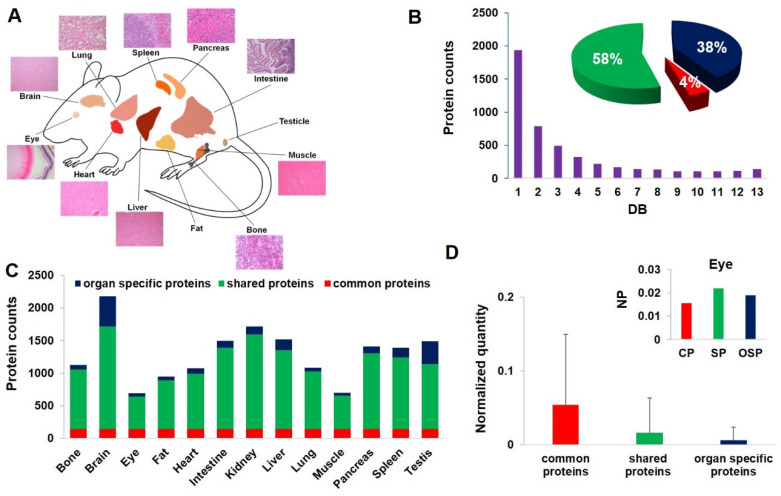
Overall results of mouse panorgan proteome. (**A**) Illustrations and histology of the organs and tissues were analyzed. (**B**) The distribution of detected proteins as a function of detection breadth, i.e., the number of organs/tissues in which a protein was detected. The insert shows the percentage of common proteins (DB = 13), shared proteins (DB = 2~12), and organ-specific proteins (DB = 1) in the total proteome. (**C**) The number of proteins detected per organ/tissue. (**D**) The averaged and normalized detection quantity of the three groups of proteins. The error bar is the standard deviation. The insert is the protein detection quantity in the eye, in which NP is normalized quantity, CP is common proteins, SP is shared proteins, and OSP is organ-specific proteins.

In our study, we applied shotgun proteomics and characterized relatively abundant proteins from 13 major organs in C57BL/6J (BL6) mice, as denoted in Figure 1A, i.e., the brain, spleen, eye, bone, fat, lung, heart, kidney, liver, intestines, pancreas, testis, and muscle. We then examined the selected organ proteomes from three other in-bred mouse strains, including A/J, NOD/ShiLtJ (NOD), and SJL/J (SJL). All the results were deposited in PeptideAtlas (http://www.peptideatlas.org (PASS01713, accessed on 30 November 2021 )).

To be practical, we reduced the analysis depth by avoiding additional offline fractionations and only used a single LC-MS/MS injection for each organ. Such analysis only detects relatively abundant proteins but allowed us to achieve a desired organ and strain coverage with reasonable instrumental time. Compared to the in-depth studies, we only identified 4787 protein groups from 163 MS runs (Supplementary Table S1). This result is far fewer than the 7349 proteins detected by Geiger et al. from 28 tissue types of BL6 [9] with deep fractionation and a total of 336 MS runs. However, we were able to extend the proteome coverage from BL6 to three more genetic backgrounds. Such expansion was crucial for us to evaluate the variability of organ-specific proteins (OSPs).

### 3.2. Protein Distribution across Tissues

We used the detection breadth (DB), i.e., number of organs/tissues from which a particular protein was detected, to describe the detection distribution. Figure 1B summarizes the results, in which the number of identified proteins displays a concave shape as a function of DB. This trend is consistent with reported studies on human and mouse proteomes and transcriptomes [7,9,35]. Using the DB value, we defined three types of proteins, i.e., organ-specific proteins (OSPs) (DB = 1), common proteins (DB = 13), and shared proteins (DB = 2–12). The percentage of proteins in each category to the total proteome is shown in the Figure 1B insert. When the total identification was broken down to individual organs, as shown in the bar chart in Figure 1C, the brain and the eye had the highest and lowest numbers of detected proteins, respectively, which was consistent with previous reports [36]. We estimated protein expression by normalized spectra counting [32]. Common proteins have, on average, higher expression than shared proteins and OSPs, as shown in Figure 1D. When breaking it down to individual organs, the trend is the same except for the eye, as shown in the Figure 1D insert. For the eye, many OSPs such as crystallin A, are dominant and show higher expression than abundant common proteins, such as alpha collagen, beta actin, and alpha enolase, suggesting that OSPs are not necessarily always in low abundance. It is therefore important to catalog OSPs for their individual characteristics.

### 3.3. Organ Specificity of Proteins

As we introduced, organ specificity is relative to the detected proteins, and the search for absolute organ specificity is currently impractical. However, with consistent analysis depth and a uniform sample-processing scheme, we can define relative organ specificity or conditional specificity. Because of our relatively shallow penetration in the proteome compared to the depth study, our detection of leakage expression was less severe. As a result, more OSPs are defined in our dataset. For example, 1602 OSPs were derived from BL6 that occupied 40% of the total identified proteins, whereas only 43 proteins (1.1% of total identification) were commonly detected in all organs examined. This result contrasts with those of depth studies. Specifically, in the recently mapped human proteome, more than 2350 proteins (~13.6%) [7] were detected as common; in the BL6 mouse proteome, 1972 proteins (27%) [9] were detected ubiquitously, and only 650 proteins (8.8%) were from one tissue. Clearly, higher penetration in the proteome expanded the pool of common proteins, even though many of them were dominant in only one or a subset of tissues. By sampling relatively abundant proteins, we effectively avoided the detection of leakage proteins that are ubiquitously present in low abundance, which helped to increase the OSP population. However, shallow penetration will miss many critical low-abundance proteins that are also organ-specific, such as surface proteins and transcription factors.

Next, we studied how these OSPs change in different genetic backgrounds. For this, we extended the analysis in BL6 to the additional three strains, i.e., A/J, SJL, and NOD. The total trend in terms of the percentage of OSPs and common proteins to the respective total proteome of each strain was very similar. When we compared the panorgan proteomes among the four strains, the *D* score was 0.25. When we compared the OSPs, however, the *D* score was 0.63, which was much higher than the overall proteome. This observation suggests that OSPs are more sensitive to the diversity of genetic backgrounds than the total proteomes.

Taking advantage of the multigenetic backgrounds, we filtered our OSPs for their robustness. First, we excluded any OSPs that were designated to different organs/tissues among the four strains, which were more frequent in organs that are closely associated, such as the heart and the muscle. Next, we scored the rest of the OSPs with an R value, i.e., the frequency of detecting the same organ specificity among the four strains. The maximum R value was 4, and we filtered the OSPs with R ≥ 2. A criterion indicates an OSP needs to be verified in at least two out of four mouse strains. A total of 655 proteins passed the filtering, and we named them verified OSPs (vOSPs), as listed in Supplementary Table S2. This included 467 proteins with R = 2, 151 proteins with R = 3, and 37 proteins with R = 4.

**Figure 2:**
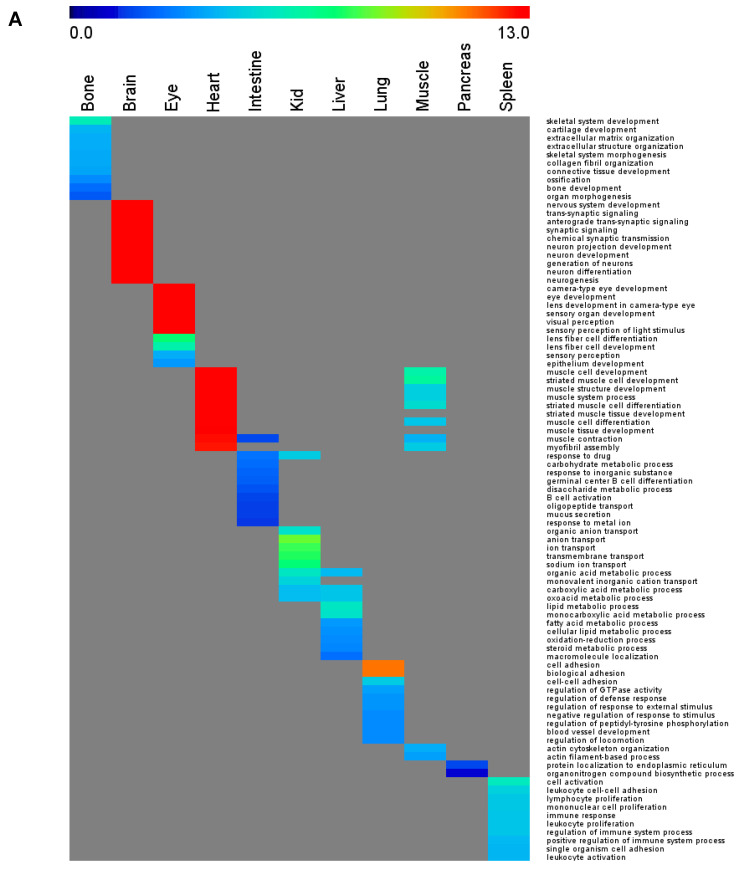

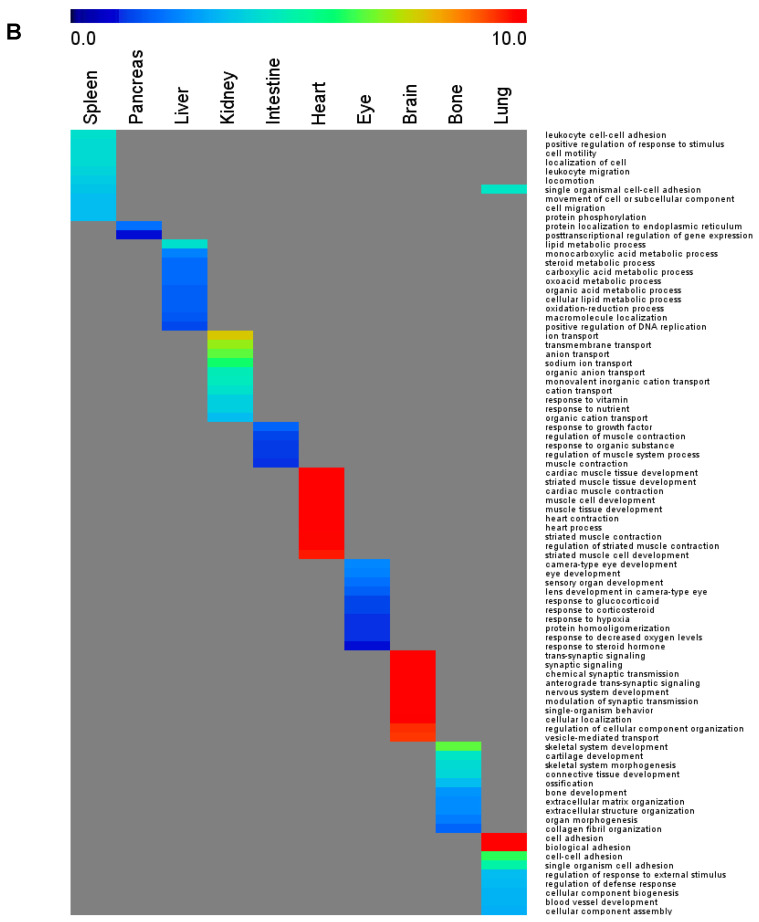

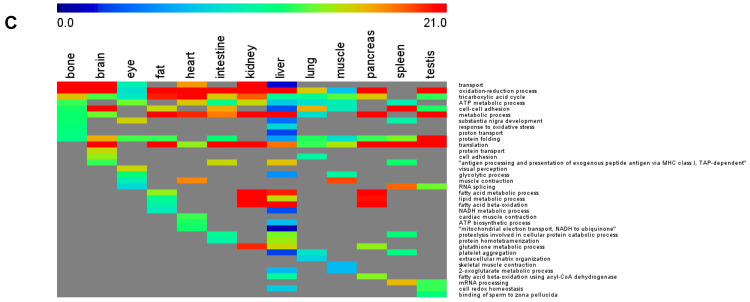
Gene ontology enrichment analysis of biological processes in the obtained protein lists. (**A**) Top10 enriched biological processes of filtered and partially verified organ-specific proteins (vOSPs) of each organ against four genetic backgrounds. (**B**) Top10 enriched biological processes of complete OSPs of each organ across four genetic backgrounds. (**C**) Top10 enriched biological processes of each organ proteome of BL6.

To compare the biological significance of the vOSPs from our breadth study to depth studies, we analyzed the functions enriched by vOSPs. Figure 2A and Supplementary Table S3 summarize the top 10 most enriched biological processes obtained by DAVID Bioinformatics Resources [34]. It is clear that the enriched biological processes can well represent organ functions, an observation that is consistent with those of depth studies [8,9]. We further examined the organ specificity of the enriched biological processes. The *D* score for the enriched biological processes was 0.84, suggesting high organ specificity. Some of these characteristic functions are listed here. In the eye vOSPs, “eye development”, “visual perception”, and “lens fiber cell differentiation” were top hits; those for brain vOSPs included “nervous system development”, “synaptic signaling”, and “neurogenesis”; those for bone vOSPs included “skeletal system development”, “bone development”, “cartilage development”, “extracellular matrix organization”, and “ossification”; those for the lung comprised “cell adhesion”, “regulation of defense response”, “regulation of response to external stimulus”, and “blood vessel development”; those for the heart mostly shared with those from the muscle including “muscle cell development”, “striated muscle development”, and “muscle system processing”; those for the kidney included “ion transport” and “transmembrane transport”. Several metabolic processes were enriched in the vOSPs of both the kidney and the liver, such as “organic acid metabolic process”, “carboxylic acid metabolic process”, and “oxoacid metabolic process”. For liver vOSPs, the uniquely enriched biological processes were “lipid metabolic process”, “monocarboxylic acid metabolic process”, and “fatty acid metabolic process”. The only organ we did not verify was the testis, as it has a relatively large number of unique OSPs, as previously reported [35], which can be reflected by their enriched GO terms: “sexual reproduction” and “gamete generation”. We did, however, use the testis proteome to eliminate OSPs of other organs, as it expresses vast numbers of different proteins.

We further examined those vOSPs with R score < 4. These are vOSPs that were verified in more than or equal to two strains but were absent from one or two of the other strains. The reason this occurred is because some proteins were detected in more than one organ in a particular strain; therefore, they were absent from the OSP lists of that strain. As a result, these vOSPs only maintain organ specificity in partial genetic backgrounds but not in all.

To search for OSPs that are ubiquitously organ-specific, we further filtered our vOSPs to remove these partially verified entries. In the end, only 328 OSPs remained (Supplementary Table S4), and we named them complete OSPs (cOSPs). Among them, the category of R = 4 remained the same, whereas the number of proteins with R = 3 was reduced to 99, and the number of proteins with R = 2 was reduced to 192. A similar functional enrichment analysis showed the highest organ specificity among all the datasets we examined, with a *D* score of 0.99. The results are summarized in Figure 2B and Supplementary Table S5, in which only one term overlapped between the spleen and the lung, whereas all the other terms were unique to their represented organs. Such filtering can effectively remove overlaps between closely associated organs such as the heart and the muscle, as shown in Figure 2A. However, the high stringency renders some organs unable to resolve any cOSPs or only a few. For example, the muscle has only three proteins in cOSPs, including junctophilin-1 (Jph1), a member of the junctional membrane complexes that is expressed specifically in the triad of skeletal muscle in young mice linking the transverse tubule with sarcoplasmic reticulum membrane [37,38]. The ectopic expression of Jph1 in the mouse heart caused abnormalities in junctional membrane structure [39], suggesting its muscle specificity.

### 3.4. Effects of Detection Depth and Breadth to the OSPs

To verify whether the observed tissue specificity in OSP functions was also represented in the total proteome of each organ, we ran similar enrichment analysis to panorgan proteomes. The top 10 enriched biological processes from each organ of BL6 are summarized in Figure 2C. The inclusion of common and shared proteins largely shifted the pattern of enriched GO terms away from those of OSPs (Figure 2A,B). The similarity in biological processes across organs was greatly increased, whereas organ specificity was diminished. The *D* score of the obtained biological processes was only 0.22, whereas the *D* score of those OSPs was 0.88, a 3-fold increase in organ specificity among the biological processes in OSPs compared with the panorgan proteomes. Several GO terms, such as “metabolic process”, “ATP metabolic process”, “translation”, and “protein folding”, were broadly shared in all organs.

As previously mentioned, compared to depth studies, our breadth study was not effective in identifying trace proteins in all organs. As a result, our data have more OSPs and fewer common proteins. Such shallow penetration of the panorgan proteome can introduce fewer authentic OSPs, yet our results demonstrated that filtering across strains can effectively eliminate some of these false positives. The above-addressed GO enrichment analysis showed that the filtered OSPs can well reflect on organ-specific functions. Further comparisons of the enrichment analyses among total proteomes, vOSPs, and cOSPs suggest that functional enrichment results are sensitive. The inclusion of common or shared proteins can skew results, and our *D* score can capture such skewing quantitatively.

Another drawback of shallow penetration is to miss some of the extremely low abundance, yet organ-specific, proteins. To evaluate the potential outcome of this effect, we examined our top100 proteins in each organ. The reason for analyzing this proteome is because previous depth studies have demonstrated that this proteome, even though abundant, can effectively delineate differences in organ and sample types [8,9], suggesting that such a proteome has sufficient organ-specific features that can be used to establish organ or sample identity. On the one hand, because these proteomes are rich in abundant proteins, studying such proteomes will reflect on the potential issues linked to a shallow sampling. On the other hand, because many proteins in this list are widely detected in multiple organs, it can also reflect on the issues in depth studies in which common proteins prevail.

**Figure 3:**
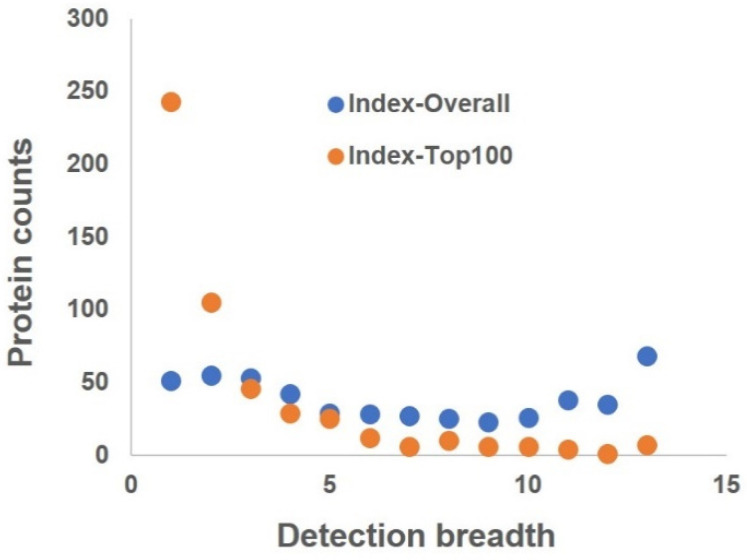
Protein distribution as a function of detection breadth in the organ proteomes, in which the Index-Overall represents the frequency a protein was detected in the overall panorgan proteome, and Index-Top100 represents the frequency a protein was in the panorgan proteomes of top100 proteins.

We analyzed the top100 proteins from each organ for OSPs similarly to how we analyzed the panorgan proteomes. Considering that the top100 proteins in each organ are more commonly detected and shared in multiple organs, we filtered the results for conditional OSPs that were dominantly detected in one organ and less frequently detected in the remaining organs. This criterion was relaxed compared to how we derived the overall OSPs, in which the proteins were filtered by BD = 1. Specifically, we created two indices for analyses. One was to index the frequency a protein was present in the top100 lists—“Index-Top100”. The other was to index the frequency a protein was present in the overall organ proteomes—“Index-Overall”. Figure 3 shows both indices as a function of distribution breadth (DB). In the figure, Index-Top100 recapitulates the overall protein distribution in Figure 1B, whereas Index-Overall shows a more even distribution than Index-Top100. The fatter overall distribution suggests that these proteins are widely shared across organs; however, most of them are not the top100 in most organs, as suggested by their value in Index-Top100. This observation suggests the possibility of deriving OSPs from Top100 proteomes, as they still carry organ differences regardless of their relatively high abundance.

To derive OSPs from the proteomes of Top100 proteins, we used Index-Top100 = 1 as the criterion to filter the list. To ensure that these proteins were dominantly detected in only one organ, we further filtered the results based on the detection quantity difference between the dominant organ and the rest, which we named “dominance fold”. Using dominance fold ≥ 4 as the cutoff, we obtained the conditional OSPs from proteomes of Top100 proteins of all four strains. Next, we examined the protein variation across the strains. Using the R scoring system, we filtered the data similarly by R ≥ 2 and manually verified the obtained proteins for single-organ dominancy and for same-organ consistency across genetic backgrounds. In the end, a total of 79 conditional OSPs from the top100 lists were obtained (Supplementary Table S6), in which 26 proteins were R = 2, 32 proteins were R = 3, and 21 proteins were R = 4.

The conditional OSPs obtained from top100 proteins were compared with vOSPs and cOSPs obtained from the overall proteomes. Clear distinctions exist. First, conditional OSPs are much more abundant in measured quantity than vOSPs and cOSPs (~2–3-fold higher). Second, there were fewer proteins from conditional OSPs than from vOSPs (>7-fold reduction) and cOSPs (>3-fold reduction). Third, the enriched biological processes were not as distinct as those of vOSPs and cOSPs. Fourth, owing to the more common detection among organs, the derivation of conditional OSPs is more challenging and requires more manual evaluation, as described above. Out of the 79 conditional OSPs from the proteomes of Top100 proteins, 43 of them were also part of the vOSPs, but only 15 of them were cOSPs. This result suggests that to obtain an adequate number of complete OSPs, a certain sampling depth is necessary; however, when the sampling depth becomes too high, the method identifies more leakage expression, which can complicate statistical analysis and exponentially increase the cost and resources spent by the experiments.

In addition to distinctions, some similarities were also revealed when we compared the three filtered lists of OSPs, i.e., vOSPs, cOSPs, and conditional OSPs from the Top 100 proteins against four genetic backgrounds. First, all three filtered OSPs were more abundant in the average detection quantity than the unfiltered lists. For vOSPs and cOSPs, such an increase ranged from 3% to 58% in four strains; for conditional OSPs, the increase ranged from 25% to 127%. This result suggested that random and erroneous detections with low quality could be effectively removed. Second, all filtered OSPs exhibited strain diversity even though much less than their unfiltered counterparts. The *D* scores of vOSPs, cOSPs, and conditional OSPs of the Top 100 were 0.24, 0.19, and 0.12, respectively, whereas the *D* score of the unfiltered OSPs (DB = 1) of the total organ proteomes were 0.63 (63%) and 0.48 (48%) for the unfiltered Top100 (Index of Top100 = 1). This result matches a recent study of the liver proteome across a large population of outbred mice, in which 50% protein diversity was observed among 192 mice [13]. Our observation suggests that the protein diversity caused by genetic backgrounds is substantial and ubiquitous even for abundant proteins, and caution should be kept when using proteins for organ-specific applications.

### 3.5. Organ-Specific Functions

**Figure 4:**
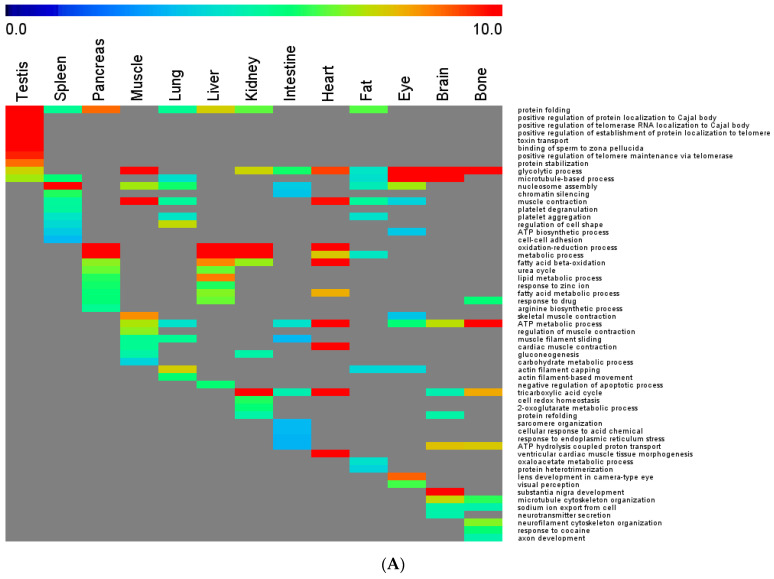

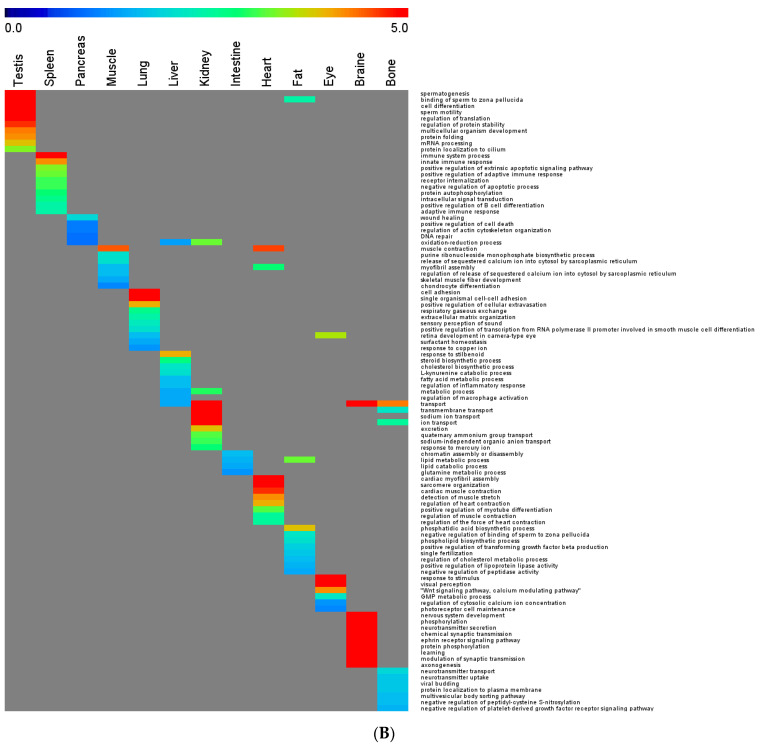
Gene ontology enrichment analysis of biological processes in the panorgan proteome of BL6. (**A**) Enrichment analysis of the pan-organ proteomes of top100 proteins. (**B**) Enrichment analysis of the organ-specific proteins.

As described above, our GO enrichment analysis demonstrated that the functions derived from OSPs are also organ-specific. To further investigate the relationship between the enriched biological functions and the corresponding protein lists, we used BL6 as an example and examined its Top 10 enriched biological processes from the Top 100 proteins of each organ. Figure 4A and Supplementary Table S7 summarize the results. For comparison, the Top 10 enriched biological processes in OSPs of BL6 are also plotted in Figure 4B. It is clear that the Top 100 proteins resemble the panorgan proteomes for the enriched biological processes, as shown in Figure 2C, in which shared GO terms across tissue types are common but dissimilar to those of OSPs in Figure 2A,B and Figure 4B. The trend can also be well reflected by the *D* scores across different organs. The *D* score of the enriched biological processes for the Top100 of BL6 was 0.34; those for the total proteome and OSPs of BL6 were 0.22 and 0.88, respectively. The higher *D* score in the Top100 proteomes than in the total proteomes suggests that organ-specific functions are also represented by abundant proteins in addition to low abundance proteins.

In our studies, the agreement between the enriched GO terms and the corresponding protein lists was not always the same. From our past study of housekeeping genes [40], a disagreement stood out. In that study, we discovered that in 13 published housekeeping gene lists by pan-human organ/tissue transcriptomics analyses, there was only a single gene in common. However, the corresponding enriched GO biological processes were surprisingly uniform and consistent. We advocated “housekeeping functions” over “housekeeping genes” [40] due to the unanimity of the enriched biological functions and the disparity in the identified housekeeping genes. Even though in sharp contrast to the GO terms derived between the housekeeping genes and the OSPs, as we showed here, both results suggest that gene ontology can well reflect the overall state of the identified proteins and can be used as a reliable criterion to evaluate the nature of the obtained transcripts and proteins.

## 4. Conclusions

Here, we present a new experimental scheme to study organ-specific proteins (OSPs) that we named the breadth study. In this scheme, we expanded the coverage of diverse genetic backgrounds balanced by decreased analysis depth as opposed to the existing in-depth studies. Our results revealed large diversity in OSPs in mice of different genetic backgrounds, and we used the robustness score (R) to catalog OSPs that are consistent across genetic backgrounds from those that are varied. Depending on the sampling depth and the number of OSPs and their derivation process, their robustness can vary substantially. Nevertheless, at all abundance levels, filtering OSPs for consensus among genetic backgrounds by the breadth study can effectively purify OSPs. Furthermore, we discovered that biological functions enriched by the corresponding OSPs are also organ-specific and sensitive to the proteins from which they are derived. We defined the *D* score here to quantitatively evaluate organ specificity at both the protein level and functional level. A higher *D* score indicates a higher organ specificity and less overlap to other organs.

Finally, linking our observed diversity in mouse strains and those made in gene expression analyses of eQTLs and pQTLs [13,14,15] to the clinical knowledge of organ-specific biomarkers in patients [19,41], we emphasize the importance of addressing such dynamic changes in OSPs in a population. The analysis here demonstrated that a single nanoLC-MS/MS run carried out by modern high-accuracy and high-sensitivity tandem mass spectrometry within 1–2 h can provide sufficient protein identifications to enable fast and practical breadth studies. It allows the derivation of a relatively large number of OSPs to quantitatively distinguish them into relatively small subcategories based on their R and *D* scores for various clinical as well as biomedical applications. Compared with the existing depth studies, the breadth analysis extends an orthogonal dimension with comparable resources, simplified statistics, and a shortened distance to clinical utility. More importantly, such a study can be easily scaled to include more strains and conditions due to its label-free nature. We hope the strategy and the lists of the filtered OSPs can benefit medical and biological research in organ-specific diagnoses and treatments.

## Data Availability

Peptideatlas.org/PASS01713.

## Supplementary Materials

The following are available online at https://www.mdpi.com/article/10.3390/cells10123449/s1.
