# Supplementary material for: Mouse Organ-Specific Proteins and Functions
Source: Cells. 2021 Dec 8;10(12):3449. doi: 10.3390/cells10123449 (PMC8700158; doi:10.3390/cells10123449)
Supplement: Supplementary file 1 [file cells-10-03449-s001.zip › supplementary/OSP_supplementary.pdf]

**SUPPLEMENTARY MATERIALS:**

# Mouse Organ Specific Proteins and Functions

B. Sun[1*], C. Lorang[2], S. Qin[2], Y. Zhang[1], K. Liu[1], G. Li[2], Z. Sun[2], A. Francke[1], A. G. Utleg[2], Z. Hu[2], K. Wang[2], R. Moritz[2], and L. Hood[2*]

**Affiliations:**

[1] Simon Fraser University, Burnaby, BC, Canada.

[2] Institute for Systems Biology, Seattle, WA, USA.

*Correspondence to: Bingyun Sun, email: bingyun_sun@sfu.ca; Leroy Hood, email: lhood@systemsbiology.org.

## TABLE OF CONTENTS

**Methods**

1. Tissue histology

2. Proteomic sample processing

3. MS analysis of peptides.

4. MS/MS database search parameters and protein inference

**Supplementary Tables**

**Table S1:** Total detected proteins.

**Table S2:** Verified Organ Specific Proteins (vOSPs).

**Table S3:** Top10 enriched biological processes of Verified Organ Specific Proteins.

**Table S4:** Complete Organ Specific Proteins (cOSPs)

**Table S5:** Top10 enriched biological processes of Complete Organ Specific Proteins.

**Table S6:** Conditional OSPs from top100 proteins.

**Table S7:** Top10 enriched biological processes of conditional OSPs.

**Methods:**

*Tissue histology.* Sections of excised organs were stored in 10% formalin for H&E staining in the Department of Pathology, University of Washington, Seattle, WA. General steps included embedding of tissues in paraffin, slicing and mounting on microscope slides, and staining with Hematoxylin and Eosin (H&E staining) prior to microscopic imaging.

*Proteomic sample processing.* Frozen organs were pulverized in a ceramic crucible chilled with liquid nitrogen and collected in Eppendorf tubes. PBS with a 1:100 diluted protease inhibitor cocktail (PIC) was used to rinse tissues on ice until the majority of blood had been removed. Then, samples were transferred to PBS buffer with 1:100 PIC and 2-5% SDS before being homogenized by Precellys24 (Bertin, France) at 4 °C. The supernatant was obtained by centrifugation, and the amount of proteins was quantified by BCA. Fifty microliters of samples were processed for digestion. In detail, TCEP and EDTA were introduced to final concentrations of 10 mM and 5 mM, respectively. Samples were boiled at 100°C for 10 min at first and then processed based on the filter aided sample preparation (FASP) method [1], in which 8 M urea was introduced and SDS was removed by filtration. Denatured and reduced proteins were alkylated by sodium iodoacetamide and quenched by DTT before sequence grade trypsin at a 1:50 enzyme to protein ratio was introduced to digest the sample at 37 °C overnight. The digested samples were desalted on Sep-Pak C18 columns and dried in a SpeedVac® (Thermo Savant, Holbrook, NY, USA) concentrator.

*MS analysis of peptides.* Three different systems were used, i.e. LTQ XL linear ion trap mass spectrometer (LTQ), Velos Pro dual-pressure linear ion trap mass spectrometer (Velos), and LTQ-Orbitrap XL hybrid ion trap-Orbitrap mass spectrometer (Orbi). For LTQ analysis, similar

to previously published procedures [2,3], an in-house fabricated nanoelectrospray source and an HP1100 solvent delivery system (Agilent Technologies) were coupled to the tandem MS. Samples were automatically delivered by a FAMOS autosampler (LC Packings, San Francisco, CA) to a 100-µm (ID) fused silica capillary precolumn packed with 2 cm of 200-Å pore size Magic C18AQTM material (Michrom Bioresources, Auburn, CA). Samples were washed with solvent A (5% acetonitrile in 0.1% formic acid) on the precolumn, eluted with a gradient of 10–35% solvent B (100% acetonitrile) over 60 min to a 75 µm x 10 cm fused silica capillary column packed with 100-Å pore size Magic C18AQ material (Michrom Bioresources), and then injected into the MS at a constant flow rate of 300 nL/min. Eluting peptides were analyzed by tandem MS in data-dependent acquisition, in which five most abundant precursor ions were selected for MS2 fragmentation with a dynamic exclusion of 1 repeat count in 30 sec for 180 sec duration, and the size of the exclusion list was 50. For Orbi analysis, an Agilent 1200 HPLC was coupled to the instrument. Peptides were applied to a fused and fritted silica capillary precolumn of 100-µm ID (New Objectives, Woburn, MA) packed with 2 cm of 200 Å pore-size C18 resin. Samples were subsequently washed with solvent A (5% acetonitrile in 0.1% formic acid) on the precolumn, eluted with a gradient of 10-35% solvent B (100% acetonitrile) over 60-90 minutes to a 75 µm x 10 cm fused silica capillary column packed with 100 Å pore-size Magic C18AQ™ (Michrom Bioresources, Auburn, CA), and then injected into the Orbi with a nano-ESI source. Eluting peptides were analyzed in MS by a similar data-dependent acquisition as before with a dynamic exclusion setting of 1 repeat count in 30 sec for 60-sec duration, and the size of the exclusion list was 50 (60-min gradient) or 500 (90-min gradient). Majority of samples were analyzed by Orbi method. For Velos analysis, Eksigent nano-HPLC was used with similar columns and a 60-min LC method as Orbi. Similar data-dependent acquisition was carried out by Velos with minor

modifications, in which the top 10 most abundant precursor ions were selected for MS/MS fragmentation with a dynamic exclusion setting of 1 repeat count in 30 sec for 180 sec duration, and the size of the exclusion list was 50.

*MS/MS database search parameters and protein inference.* For the Orbi and Velos runs, the MS1 mass error was set to ± 50 ppm; for the LTQ runs, it was set to ±3.0 Da. For MS2, 0.5 Th was used for all analyses. For all runs, peptides were allowed to be semitryptic with up to two internal cleavage sites. The search parameters included a fixed modification of +57.0215 for carbamidomethylated cysteines and a variable modification of +15.9949 for oxidized methionines and +42.0106 for protein n-terminal acetylations.

**REFERENCES:**


1 Wisniewski, J. R., Zougman, A., Nagaraj, N. & Mann, M. Universal sample preparation method for proteome analysis. *Nature methods* **6**, 359-362, doi:10.1038/nmeth.1322 (2009).

2 Sun, B. *et al.* N-Glycoproteome of E14.Tg2a mouse embryonic stem cells. *PLoS ONE* **8**, e55722, doi:10.1371/journal.pone.0055722 (2013).

3 Sun, B. *et al.* Shotgun Glycopeptide Capture Approach Coupled with Mass Spectrometry for Comprehensive Glycoproteomics. *Mol. Cell Proteomics* **6**, 141-149, doi:10.1074/ (2007).
